# Increasing knock-in efficiency in mouse zygotes by transient hypothermia

**DOI:** 10.1089/crispr.2023.0077

**Published:** 2024-04-01

**Authors:** Amine Bouchareb, Daniel Biggs, Samy Alghadban, Christopher Preece, Benjamin Davies

**Affiliations:** 1 Wellcome Centre for Human Genetics, Oxford, UK, OX3 7BN; 2 The Francis Crick Institute, London, UK, NW1 1AT

**Keywords:** Gene editing, zygote, electroporation, homology directed repair

## Abstract

Integration of a point mutation to correct or edit a gene requires the repair of the CRISPR-Cas9-induced double-strand break by homology directed repair (HDR). This repair pathway is more active in late S and G2 phases of the cell cycle, whereas the competing pathway of non-homologous end joining (NHEJ) operates throughout the cell cycle. Accordingly, modulation of the cell cycle by chemical perturbation or simply by the timing of gene editing to shift the editing towards the S/G2 phase has been shown to increase HDR rates.

Using a traffic light reporter in mouse embryonic stem cells and a fluorescence conversion reporter in human induced pluripotent stem cells, we confirm that a transient cold shock leads to an increase in the rate of HDR, with a corresponding decrease in the rate of NHEJ repair. We then investigated whether a similar cold shock could lead to an increase in the rate of HDR in the mouse embryo.

By analysing the efficiency of gene editing using SNP changes and loxP insertion at 3 different genetic loci, we found that a transient reduction in temperature after zygote electroporation of CRISPR-Cas9 ribonucleoprotein with an ssODN repair template did indeed increase knock-in efficiency, without affecting embryonic development. The efficiency of gene editing with and without the cold shock was first assessed by genotyping blastocysts. As a proof of concept, we then confirmed that the modified embryo culture conditions were compatible with live births by targeting the coat colour gene Tyrosinase and observing the repair of the albino mutation. Taken together, our data suggest that a transient cold shock could offer a simple and robust way to improve knock-in outcomes in both stem cells and zygotes.

## Introduction

Genetically altered animal models provide invaluable insights into human biology, disease pathology, and allow therapeutic approaches to be tested and optimized. In the mouse, targeted gene manipulation has been routine for several decades, originally achieved using homologous recombination in embryonic stem (ES) cells to remove or replace sequences ^1^. More recently, the emergence of site-specific DNA endonucleases such as Zinc Finger Nucleases (ZFN) ^2^, Transcription Activator-Like Effector Nucleases (TALENs) ^3^ and the CRISPR-Cas RNA-dependent nucleases ^4–6^, has increased the efficiency of targeted manipulation, allowing direct manipulation of the zygote.

The CRISPR-Cas9 system, in particular, has become the technology of choice for genetic alteration due to its simplicity of design ^7^. However, one of the challenges of using nucleases for the generation of targeted replacement or knock-in mutations are the competing repair mechanisms that operate to process the nuclease-induced double strand break. The repair pathways most active in the mammalian cell are non-homologous end joining (NHEJ) and microhomology-mediated end joining (MMEJ) which lead to insertion and deletion (indel) mutations at the target site. Although highly useful for achieving knock-out mutations by ablating gene sequences and shifting open-reading frames, the significant repair pathways used to achieve targeted gene replacements using co-introduced homology repair templates, for example homology directed repair (HDR), operate at considerably lower efficiencies ^8^. Subsequently, following the introduction of CRISPR-Cas9 into mouse zygotes with a suitable repair template designed to achieve a targeted knock-in, most embryos undergo indel mutagenesis (via non-homology end joining (NHEJ) or microhomology-mediated end joining (MMEJ)) rather than targeted repair using the introduced template (via HDR mechanisms).

To overcome this challenge, researchers are exploring strategies to alter the balance in repair outcomes in favour of achieving the desired knock-in event. The delivery route used to deliver CRISPR-Cas9 reagents and donor template to zygotes or cells ^9,10^, the choice of the Cas nuclease ^11,12^ and the format of the repair template ^13–16^ can be optimized to significantly improve knock-in efficiency. In addition, chemical modification of the DNA repair pathways by inhibiting key components of the NHEJ and/or MMEJ pathways or promoting aspects of the HDR pathway have been shown to improve knock-in outcome in cells ^17–19^ and embryos ^20–22^.

The timing of the delivery of the Cas9 ribonucleoprotein (RNP) complex and HDR template with respect to the cell cycle has also been explored. Whilst NHEJ is active throughout the cell cycle, the HDR repair mechanism is more active during the S and G2 cell cycle phases ^23,24^. Thus, manipulating cell cycle progression by prolonging the S/G2 phases can help improve knock-in outcomes ^25,26^. Indeed, the long G2 phase in the 2-cell mouse embryo has been exploited to increase knock-in efficiency by introducing the gene editing reagents at this developmental stage ^27^.

The use of cell cycle or DNA repair pathway inhibitors on embryos might have a negative impact on embryonic development or may have other non-specific effects such as increased levels of off-target mutations. An alternative strategy reported in cells, involves no chemical intervention and is simply to induce a cold shock by reducing the temperature for a short period of time. It has been reported that this manipulation resulted in the accumulation of nucleases proteins and enhanced the mutagenesis efficiency for both ZFNs and TALENs in cells ^28,29^. Several recent studies have reported an improvement in the HDR rates using CRISPR-Cas9 in human induced pluripotent stem (iPS) cells ^30–32^. The use of CRISPR-Cas9 in combination with a cold shock has not been thoroughly investigated *in vivo*. However, Remy et al., showed a beneficial effect of a short mild hypothermic treatment on HDR-mediated transgene integration in rat zygotes injected with TALENs ^33^. Although no significant changes in targeting efficiency were reported in this study, the authors concluded that the increased trend in targeting efficiency warranted further examination. It seems likely that the cold shock could be exerting its effect through alterations to the cell cycle, but the mechanisms involved in this stimulation of gene knock-in efficiency remain unclear.

In this study, we have investigated the application of cold-shock on the efficiency of knock-in alleles when CRISPR-Cas9 machinery is introduced into the mouse zygote, exploring 3 independent gene loci. We first verified the effects of moderate transient hypothermia on repair outcomes in human iPS cells and mouse ES cells using fluorescent reporters. We then explored whether this manipulation can be applied in the mouse zygote and here report conditions that lead to a stimulation of knock-in efficiency. We demonstrate by the production of live mice that the conditions are compatible with normal subsequent mouse development.

## Materials and methods

### Cell lines and cell culture

Human iPS cells (KOLF2-C1) were cultured in Essential 8™ (E8) medium (STEMCELL Technologies, #A1517001) on tissue culture flasks coated with Vitronectin diluted per manufacturer’s instructions (Gibco™ #A14700) and with daily medium changes. For passaging, the cells were washed with PBS and treated with TrypLE™ Select (Thermo Scientific™, #12563011) or using ReLeSR™ (STEMCELL Technologies, #05872) for 5 min and cell pellets re-plated at the required density in Essential 8™ (E8) medium supplemented with 5 μM Y-27632 (Rock Inhibitor, Sigma-Aldrich).

Mouse JM8F6 ES cells were cultured in Knockout DMEM (LifeTechnologies) supplemented with 2 mM L-Glutamine, 1× non-essential amino acids, 0.1 mM β-mercaptoethanol, 1000 U/ml ESGRO (Millipore) and 10% fetal bovine serum (LifeTechnologies) on tissue culture flasks on a feeder layer of mouse embryonic fibroblasts or on plates coated with 0.1% Gelatin after electroporation. For routine passage of stem cells grown on feeders, confluent flasks were washed twice with pre-warmed PBS and trypsinized with Trypsin (0.5% Trypsin, 0.1% chicken serum, 20 μg/ml EDTA, 10 μg/ml D-Glucose in PBS) for 8 minutes at 37°C then replated at the required density in medium.

### iPS cell fluorescent reporter system

A KOLF2-C1 human iPS cell line with a single copy of a CAGGS promoter-driven GFP cassette within the *AAVS1* safe harbour locus was generated via Bxb1 recombinase mediated cassette exchange. A generic *AAVS1* targeting vector (Addgene #22075) was adapted by replacing the SA-T2A-Puromycin resistance cassette with a CAGGS promoter driving a Hygromycin-P2A-Bxb1 integrase cDNA cassette with Bxb1 attP sites flanking the open reading frame. Following gene targeting in KOLF2-C1 cells, recombinant iPS cells were transfected with a shuttle vector containing Bxb1 attB sites flanking a loxP-flanked promoterless Neomycin cassette followed by a promoterless GFP open reading frame. Expression of the Bxb1 integrase from the *AAVS1* targeted docking site catalysed the cassette exchange, resulting in neomycin resistant iPS cells with a GFP cassette integrated. The GFP cassette was then activated by transient expression of Cre recombinase (Supplementary Figure 1A), delivered by lipofection of a pCre-Pac vector ^34^.

### ES cell traffic light reporter system

A JM8F6 mouse ES cell line with a single copy of a CAGGS promoter-driven inactive mRFP1 cassette (with a STOP codon at amino-acid position 30, followed by a 27 bp deletion encompassing amino acid positions 33-41), followed by an out-of-frame P2A-eGFP cassette and a rabbit beta globin polyadenylation sequence was targeted to the *Gt(ROSA26)Sor* locus by gene targeting. A targeting vector for the *Gt(ROSA26)Sor* locus, pROSA26.10 (hygro attP) was obtained as a kind gift from Ralf Kuehn ^35^ which contained homology arms, a diphtheria toxin A chain (dtA) negative selection cassette and a PGK driven hygromycin resistance cassette. The dTA cassette and a portion of the 3’ homology arm was excised, creating a shortened targeting vector. A CAGGS promoter-driven inactive mRFP1 cassette (with the aforementioned mutations), followed by an out-of-frame P2A-GFP cassette was generated by gene synthesis and cloned between the two homology arms). Gene targeting was performed using standard conditions and recombinant clones were screened for targeted integration as previously described ^36^.

### CRISPR Reagents and delivery

CRISPR guide RNAs were designed using the CRISPOR algorithm (http://crispor.tefor.net). The guide sequences were ordered from Synthego as chemically modified single guide-RNAs (sgRNA). Alt-R^®^ S.p. HiFi Cas9 Nuclease V3 was obtained from IDT. The HDR templates were designed as 135 nt single-stranded oligodeoxynucleotides (ssODN) with homology arms flanking sequences on both sides of the cut-site. Silent mutations were introduced within the PAM sites to prevent recutting. For the ssODN designs used for the in-vivo study, an extra silent mutation was introduced within the guide RNA recognition sequence to create a diagnosis restriction site to simplify genotyping.

Cas9 protein (890 nM) / sgRNA (1.48 μM) were delivered as ribonucleoprotein complexes with ssODN (3.7 μM) to 1x10^5^ cells (human iPS or mouse ES) using the ThermoFisher Neon Electroporation System (10 μl) (1200V, 30ms, 2 pulses). Immediately following the electroporation, the cells were either incubated at 30°C for 24h followed by 48h at 37°C or were incubated at 37°C for 72h in a 5% CO_2_ incubator. At 72h post-electroporation, cells were detached using Trypsin (ES) or TrypLE™ Select (iPS) and analyzed with a BD LSRFortessa™ X-20 Cell Analyzer (BD Biosciences).

Sequences of the sgRNA, ssODN donors and the genotyping primers used in the study are listed in Supplementary Tables 1 and 2.

### Zygote electroporation

3-week old wild-type C57BL/6J or albino B6(Cg)-Tyr^c-2J^ /J female mice (Charles River) were superovulated and mated with wild-type or albino C57BL/6J studs. Fertilized oocytes were prepared from plugged females and up to 100 embryos were electroporated in Opti-MEM media (ThermoFisher Scientific) containing 130 ng/μl sgRNA and 650 ng/ul Cas9 protein (Alt-R^®^ S.p. HiFi Cas9 Nuclease V3, IDT). ssODN templates for homology directed repair were added to the electroporation mix at a final concentration of 430 ng/μl. Electroporation was performed with the previously described conditions ^9^. The electroporation was performed between 22 and 24 h after the hCG injection. Electroporated zygotes were either cultured overnight at 37°C or immediately cultured for 6 hours at 30°C followed by 37°C to the two-cell stage in EmbryoMax Advanced KSOM (Merck) and surgically implanted into recipient pseudopregnant CD1 females, or were cultured in vitro in KSOM-AA medium until blastocyst stage.

### Genomic DNA extraction and PCR amplification of edited regions

In vitro cultured blastocysts were lysed using standard conditions and crude lysate DNA was used to amplify the region of interest. The target gene was amplified using the primers listed in Supplementary Table 1. PCR products were denatured, reannealed and analysed on a 15% polyacrylamide gel to detect the formation of heteroduplexes indicative of mutations, as previously described ^9^. Restriction endonuclease digestion was used to determine successful homology directed repair and digested PCR product were analysed on a 2% agarose gel, followed by confirmatory Sanger sequencing.

### Animal work

All animal studies received ethical approval from the Clinical Medicine AWERB (Animal Welfare and Ethical Review Body) at the University of Oxford and were performed in accordance with UK Home Office Animals (Scientific Procedures) Act 1986 under project license PAA2AAE49. Mice were housed in individually ventilated cages and received food and water ad libitum. All surgery was performed under isoflurane inhalation anaesthesia using appropriate pre-surgical analgesia.

### Statistical analysis

Comparison of the knock-in efficiency for the different culture conditions in stem cells was performed using the t-test. The efficiencies of editing and HDR for the embryo study were tested using a logistic regression model. Each result was assigned a set of indicator variables to label which experimental method was used and which target gene was addressed. The experimental conditions (control or cold shock conditions) were treated as fixed-effects and the genes as random-effects. The model was then fit using the function glmer (family = binomial) from the R package lme4. The blastocyst development comparison was performed using a two-sided Fisher's exact test. Data sets were analysed and presented using Graphpad Prism software v8.4.

## Results

### Cold shock stimulates HDR in human iPS cells

We first wanted to confirm previous reports which showed the potential benefit of a transient cold shock on the rate of HDR in cells ^30–32^ in two independent cell types of different species. We designed two different strategies, both relying on fluorescence indicators to signal either the HDR or the NHEJ events to confirm this phenomenon in mouse embryonic stem (ES) cells and human induced pluripotent stem (iPS) cells.

**Figure 1:**
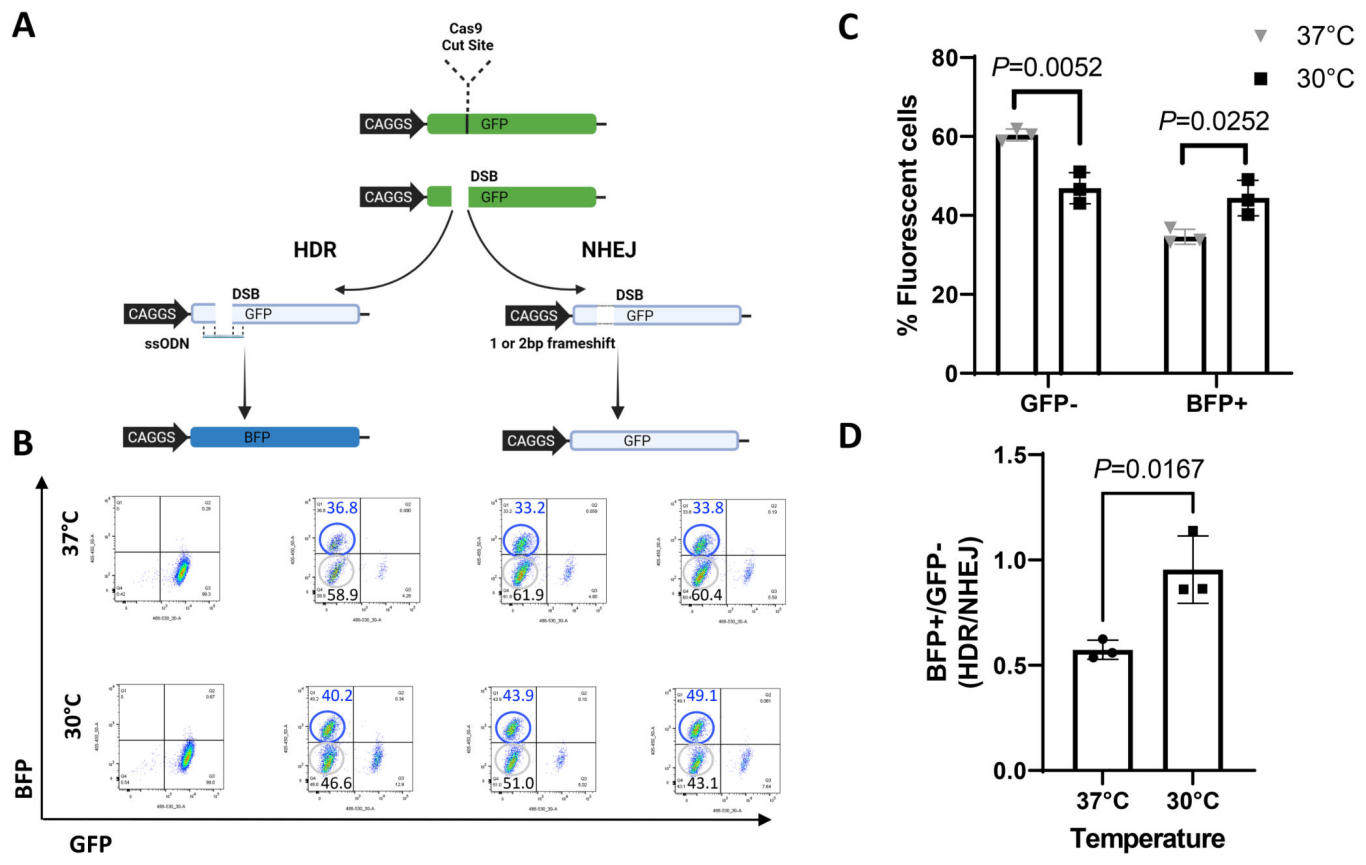
GFP to BFP assay to assess HDR in human iPS cells. **A)** Schematic representation of the GFP to BFP system. A CAGGS promoter driven GFP cassette was integrated at the *AAVS1* locus. The position of the CRISPR-Cas9 target site is shown. If the resulting DSB is repaired by NHEJ and results in a indel that shifts the reading frame, the GFP will not be translated and the cells will not fluoresce. However, if the break is precisely corrected by HDR using an ssODN to introduce a Y66H mutation, the GFP sequence will be converted into BFP, and cells will fluoresce blue. **B)** An example of an individual replicate of Flow cytometry analysis of iPS cells electroporated with CRISPR-Cas9 RNP with the ssODN and cultured for 72h at 37°C or 24h at 30°C then 48°C at 37°C. Numbers shown inside plots indicate percentages of blue or non-fluorescing cells. **C)** Histogram showing the percentage of GFP negative (GFP-) or BFP positive (BFP+) cells for the three technical replicates performed. **D)** Histogram showing the ratio of HDR/NHEJ for the three technical replicates performed.

In human iPS cells, a single copy of GFP under the control of a CAGGS promoter was integrated at the *AAVS1* locus and stable uniform fluorescence was confirmed (Supplementary Figure 1A). A gene editing strategy was adopted which aimed to convert the GFP into BFP via the incorporation of a Y66H mutation. Successful HDR would thus generate blue fluorescence, whereas disruptive indel mutations caused by NHEJ would abolish fluorescence (Figure 1A).

In order to assess the efficiency of HDR after a cold shock treatment, we electroporated the cells with Cas9/sgRNA (Supplementary Table 1) with the required ssODN (Supplementary Table 2) and cultured them, either at 37°C for 72h (control group) or 30°C for 24h then 37°C for 48h (cold shock group). Flow cytometry was used to evaluate the proportion of human iPS cells that express GFP or BFP or where fluorescence had been extinguished.

Cells incubated at 30°C for 24h showed a significantly higher GFP to BFP conversion than cells cultured without the cold shock (*P*=0.0252), suggesting a higher HDR repair frequency. Furthermore, the proportion of non-fluorescent cells due to inactivation of the GFP cassette by NHEJ-induced indel mutations was significantly higher in the cells cultured under normal condition (*P*=0.0052) than those receiving the cold shock (Figure 1B). The cold shock yielded an 28% increase in HDR efficiency (Figure 1C) and the HDR/NHEJ ratio was increased by 1.7-fold when compared to the control group (Figure 1D).

### Cold shock stimulates HDR in mouse ES cells

**Figure 2:**
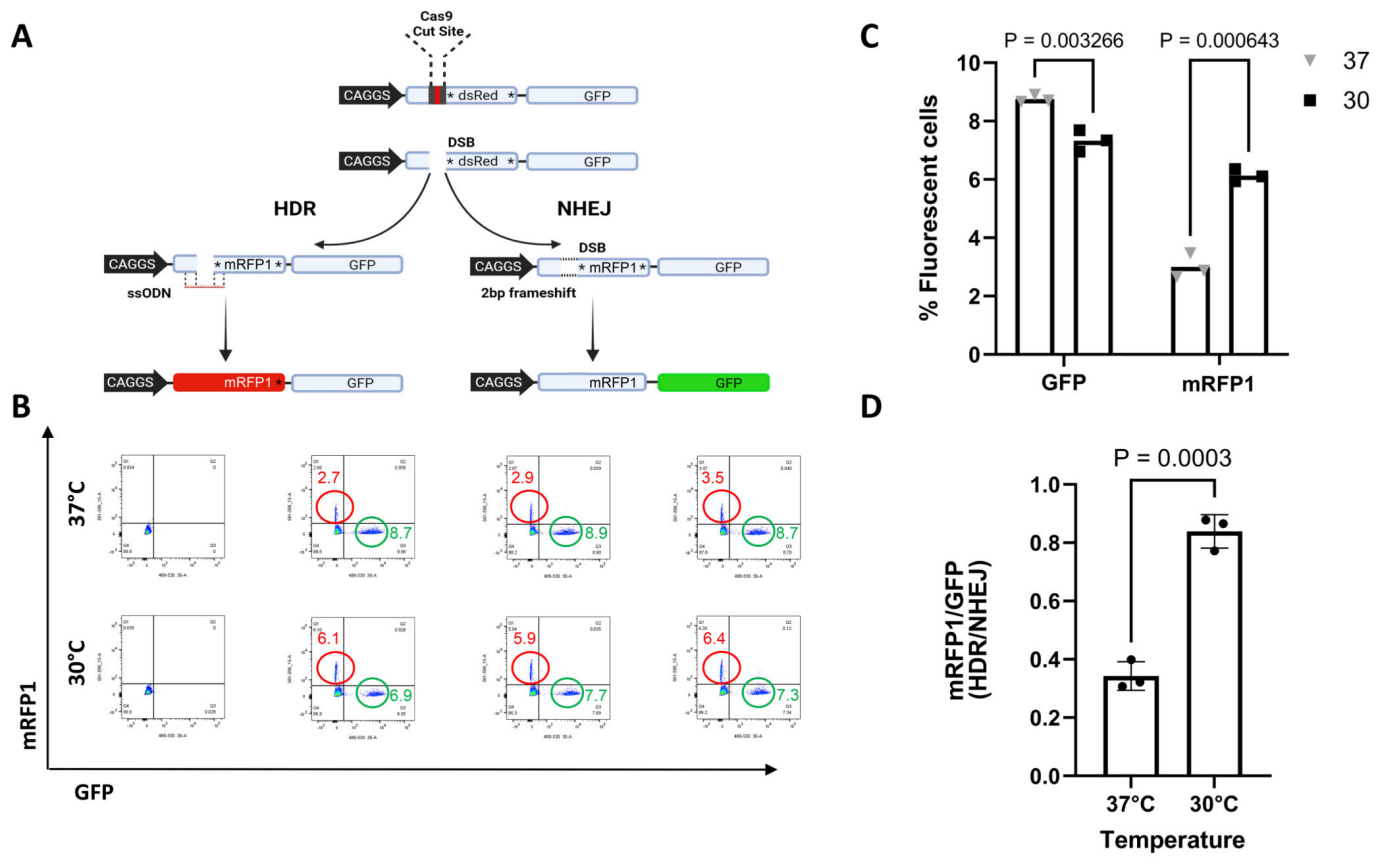
Traffic light reporter to assess HDR in mouse ES cells. **A**. Schematic representation of the TLR system. A CAGGS promoter driven mRFP1 cassette harbouring an disruptive 27 bp deletion, followed by an out-of-frame P2A-GFP cassette was integrated at the *Gt(ROSA26)Sor* locus. The position of the CRISPR-Cas9 target site is shown. If the resulting DSB is repaired by NHEJ and results in an indel that shifts the reading frame, the GFP may be brought into frame and the cells will fluoresce green. However, if the break is precisely corrected by HDR using an ssODN to repair the 27 bp deletion, the mRFP1 sequence will be restored and the cells will fluoresce red. **B)** An example of an individual replicate of Flow cytometry analysis of ES cells electroporated with CRISPR-Cas9 RNP with the ssODN and cultured for 72h at 37°C or 24h at 30°C then 48°C at 37°C. Numbers shown inside plots indicate percentages of red or green cells. **C)** Histogram showing the percentage of GFP or mRFP1 expressing cells for the three technical replicates performed. **D)** Histogram showing the ratio of HDR/NHEJ for the three technical replicates performed.

To investigate the effect of the cold shock in a different cell line, we designed a Traffic Light Reporter system ^37^ using a CAGGS driven mRFP1 cassette fused to a GFP cassette via a P2A sequence to allow bicistronic expression of the two fluorophores. The mRFP1 sequence was rendered non-functional by deleting 27 bp and additional mutations were included to ensure the downstream P2A-GFP sequence was out-of-frame. This construct was integrated at the *Gt(ROSA)26Sor* safe harbour locus of mouse ES cells (JM8F6) via gene targeting. A sgRNA was designed to target the mutated sequence of mRFP1 (Supplementary Table 1) and an 150 nt ssODN was designed to repair the deletion to restore the mRFP1 expression (Supplementary figure 1B, Supplementary Table 2). Using this strategy, mRFP1 will only result if a successful repair event has occurred via HDR, with no expression of GFP occurring due to an inframe stop codon at the end of the mRFP1 cassette. In contrast, if the repair event proceeds via NHEJ and an indel mutation is incorporated, no mRFP1 fluorescence will result but a +2 frame-shift mutation would restore the expression of the downstream GFP (Figure 2A).

In order to assess the efficiency of HDR after a cold shock treatment, we electroporated the cells with RNP with the required ssODN and cultured them, either at 37°C for 72h (control group) or 30°C for 24h then 37°C for 48h (Cold Shock Group). Flow cytometry was used to evaluate the proportion of mouse ES cells that express GFP and mRFP1.

Although, the overall editing efficiency in mouse ES cells was quite low, we observed a significant 2-fold increase in HDR efficiency for the cells that were incubated at 30°C, compared to the control cells (P=0.0006; Figure 2C). The NHEJ (using the GFP signal as a proxy) was significantly reduced by 1.2-fold for the cold shock group (*P*=0.003; Figure 2C). Moreover, their ratio of HDR/NHEJ was 2.4-fold higher when compared to the group of cells that were cultured under normal conditions (*P*=0.0003; Figure 2D).

Taking together these results confirm previously reported data on the effect of cold shock in improving precise gene repair by favouring HDR pathways over NHEJ and shows the phenomenon to be consistent in mouse stem cells.

### Cold shock stimulates HDR in mouse zygotes

Having confirmed that cold shock leads to an increase in the efficiency of knock-in allele production in mouse and human stem cells, we explored whether the effects of cold shock on HDR rate could be replicated *in vivo* by modifying the culture conditions of the mouse zygote following delivery of CRISPR-Cas9.

**Figure 3:**
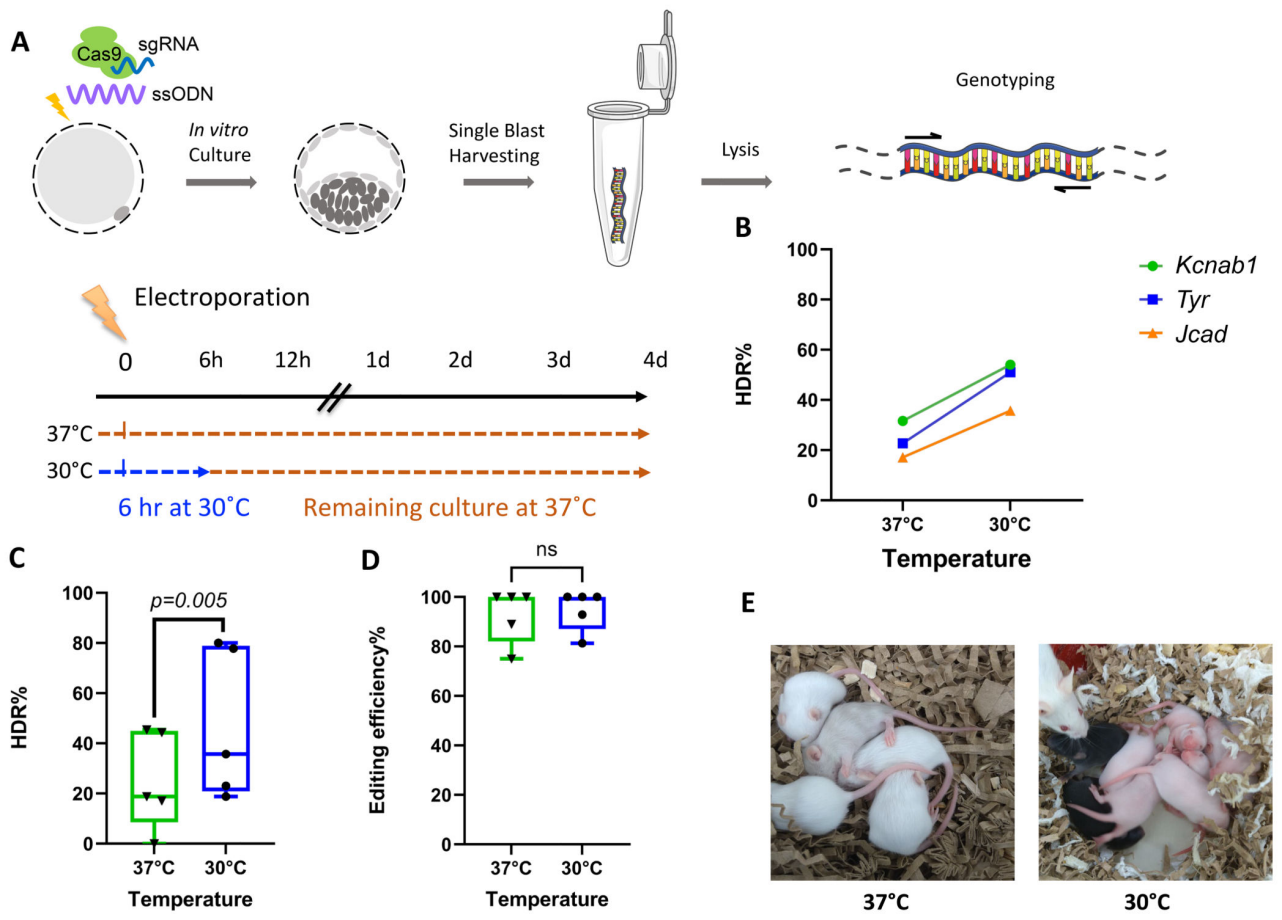
The effects of cold shock on mouse embryos. **A)** Schematic design of the knock-in experiment in mouse embryos. One-cell embryos were electroporated using CRISPR-Cas9 RNP and ssODN designed to target different loci. Embryos were cultured at 37°C for the control group or at 30°C for 6h followed by 37°C for the cold shock group. At blastocyst stage, embryos were individually harvested and genotyped. **B)** Overall HDR efficiency for the three different genes. **C)** Knock-in efficiency showing the results of the 5 independent sessions, comparing activity at the three tested genes, illustrated as a box plot (25%-75% percentile, with the whiskers extending to the minimum and maximum values. **D)** Overall editing efficiency (indel or knock-in) for the 5 independent sessions performed, as a box plot as C). **E)** Two litters of mice generated from the in vivo albino mutation correction experiment performed under control conditions (37°C) or with cold shock (30°C).

Firstly, different incubation times at 30°C were tested on cultured embryos to establish whether incubation at this unconventional temperature resulted in normal development and survival rates. Incubation of mouse embryos at 30°C for 24 hours post-harvest, did indeed result in a very low survival rate with very few embryos progressing to the 2-cell stage (data not shown). Restricting this lower temperature to a period of between 6-8 hours post-harvest resulted in normal 2-cell progression (Supplementary Figure 2A; *P*=0.058) and further embryo culture tests showed that the rate of blastocyst development was also unaffected by this duration of 30°C incubation (Supplementary Figure 2B; *P*=0.786). Further investigations revealed no difference between 6 and 8 hours of 30°C incubation (6 hours – 9.4% HDR; 8 hours – 10% HDR), therefore, we decided to limit the length of time the embryos were incubated at 30°C to 6h followed by a return to the conventional 37°C until they reached the blastocyst stage (Figure 3A).

Having established a cold shock regime which was compatible with mouse preimplantation development, we tested the effect on gene editing outcomes using sgRNAs addressing three independent gene loci (*Kcnab1, Tyr* and *Jcad*) (Supplementary Table 1) together with ssODNs to introduce mutations into these loci (Supplementary table 2). Embryos were cultured to the blastocyst stage, lysed and analysed for mutagenesis via PAGE electrophoresis and DNA sequencing (Supplementary Table 3). Culturing the embryos under cold shock conditions showed a significant improvement in HDR rate across the sgRNAs tested (β=0.985, *P*=0.005), when analysed on a gene basis (Figure 3B) or on a session basis (Figure 3C) with an average increase of in HDR efficiency of 1.9-fold compared to normal culture conditions. Notably, the overall editing (including both Indel and knock-in mutations) was not impacted by the culture conditions (β=0.417, *P*=0.491, Figure 3D).

### Production of live pups following the cold shock embryo culture conditions

Having shown a positive impact of the 6 hours cold-shock on editing efficiencies in cultured mouse embryos, in order to confirm that the cold-shock procedure did not interfere with subsequent in vivo development, we conducted a proof-of-concept in vivo study, using the Tyrosinase sgRNA and an ssODN to correct the R77L mutation responsible for the albino phenotype in B6(Cg)-Tyr^c-2J^ /J mice (Supplementary Table 1 & 2). After electroporation, embryos were cultured under normal conditions or cold shock conditions, then transferred to pseudo-pregnant females. Live pups were born from both groups, indicating that the cold-shock treatment did not adversely affect mouse development (Supplementary table 4). Furthermore, the litter size obtained in our study was comparable between groups. Although not of sufficient scale to allow a statistical analysis of the cold shock effect for live pup production, it was notable that in the litters born from the cold shock treated embryos, two entirely black pups were obtained, whereas in the litters born from the control cultured embryos, only a single mosaic pigmented pup was obtained (Figure 3E).

In summary, the results of our study confirm that the cold-shock induced augmentation of HDR efficiencies is common to both mouse and human stem cells and also preimplantation mouse embryos and the cold-shock procedure is compatible with the production of gene edited mouse models.

## Discussion

In this study, we investigated the impact of modifying the culture temperature on DNA repair pathway for Cas9-induced double strand breaks in both human and mouse stem cells and the mouse zygote. Although a number of chemical modifications have been shown to enhance HDR rate, application of such approaches to the mouse zygote may not be the preferred approach due to negative impacts on early embryonic development. Thus, a simple intervention which requires no chemical or small molecule additions to the embryo culture media was sought. Here, we wanted to test the hypothesis that, similar to the effects of transient cold shock demonstrated in cultured cells ^30–32^, such an intervention could also promote higher levels of HDR in the mouse zygote.

Studies in human iPS cells have shown that cell cycle and gene expression are affected by moderate hypothermia. Maurissen et al. found that cold shock had various effects in human iPS cells: it slowed cell-cycle progression, resulting in an accumulation of cells in G2/M phase, reduced DNA synthesis rate and enhanced the frequency of homology-directed repair ^32^. Exposure of iPS cells to 32°C for 24 or 48 hours led to a 2-fold increase in the rate of HDR ^31^. When combined with small molecule enhancer of HDR and stabilizing chemical modification of the ssODN template, an additive effect was observed favouring the HDR pathway and resulting in a seven-fold higher ratio of HDR to NHEJ ^30^.

Our results also confirm that culturing human iPS cells under cold-shock conditions (30°C for 24h) improves the efficiency of knock-in and reduces the rate of NHEJ-induced indel mutations, resulting in a 1.7-fold higher ratio HDR to NHEJ when compared to control conditions at 37°C. Similarly, when similar culture conditions were tested on mouse ES cells, we found that repair bias also shifted toward HDR over NHEJ, leading to 2.4-fold higher HDR/NHEJ ratio.

This same stimulation of HDR/NHEJ was seen in our in vivo experiments with the delivery of CRISPR-Cas9 and ssODN reagents by electroporation. It would be interesting to explore whether the transient hypothermic culture conditions slows the cell cycle progression or alters the duration of the S/G2 phase, potentially providing an explanation of why the lower temperature culture also stimulates the HDR repair, as previously shown in iPS cells ^32^.

Previous work with Zinc Finger nucleases and TALENs has explored the effects of transient hypothermia on mutagenesis rates in general. A study with zinc finger nucleases reported higher levels of mutagenesis and proposed a mechanism of reduced protein turnover of the nuclease at colder temperatures. ^28^. A similar effect of cold-shock was found for mutagenesis levels induced by TALENs. Editing rates at the *HBG1* and *HBG2* loci increased ~2-fold when human CD34^+^ peripheral blood stem cells were exposed to 30°C cold shock for 16 hours compared with 37°C culture ^38^. In contrast, we saw no overall increase in editing efficiency in our zygote and stem cell studies, with the cold-shock specifically affecting the relative proportion of repair via HDR.

Despite the interest that researchers have shown in the study of cold shock impact on gene editing outcomes in vitro using multiple cell lines, the impact of such conditions in vivo have remained poorly investigated. In summary, we have confirmed that a 6-hour cold-shock increases the efficiency of targeted mutagenesis when CRISPR-Cas9 reagents and template are delivered to the mouse zygote, whilst not impacting overall mouse development. Using this simple intervention to improve knock-in efficiencies could help reduce the animal cost of genetically altered knock-in mouse production.

## Supplementary Material

Supplementary Material
