# Supplementary material for: Increasing knock-in efficiency in mouse zygotes by transient hypothermia
Source: CRISPR J. Author manuscript; Available in PMC 2024 Apr 30. (PMC7615915; doi:10.1089/crispr.2023.0077)
Supplement: Supplementary Material [file EMS195652-supplement-Supplementary_Material.pdf]

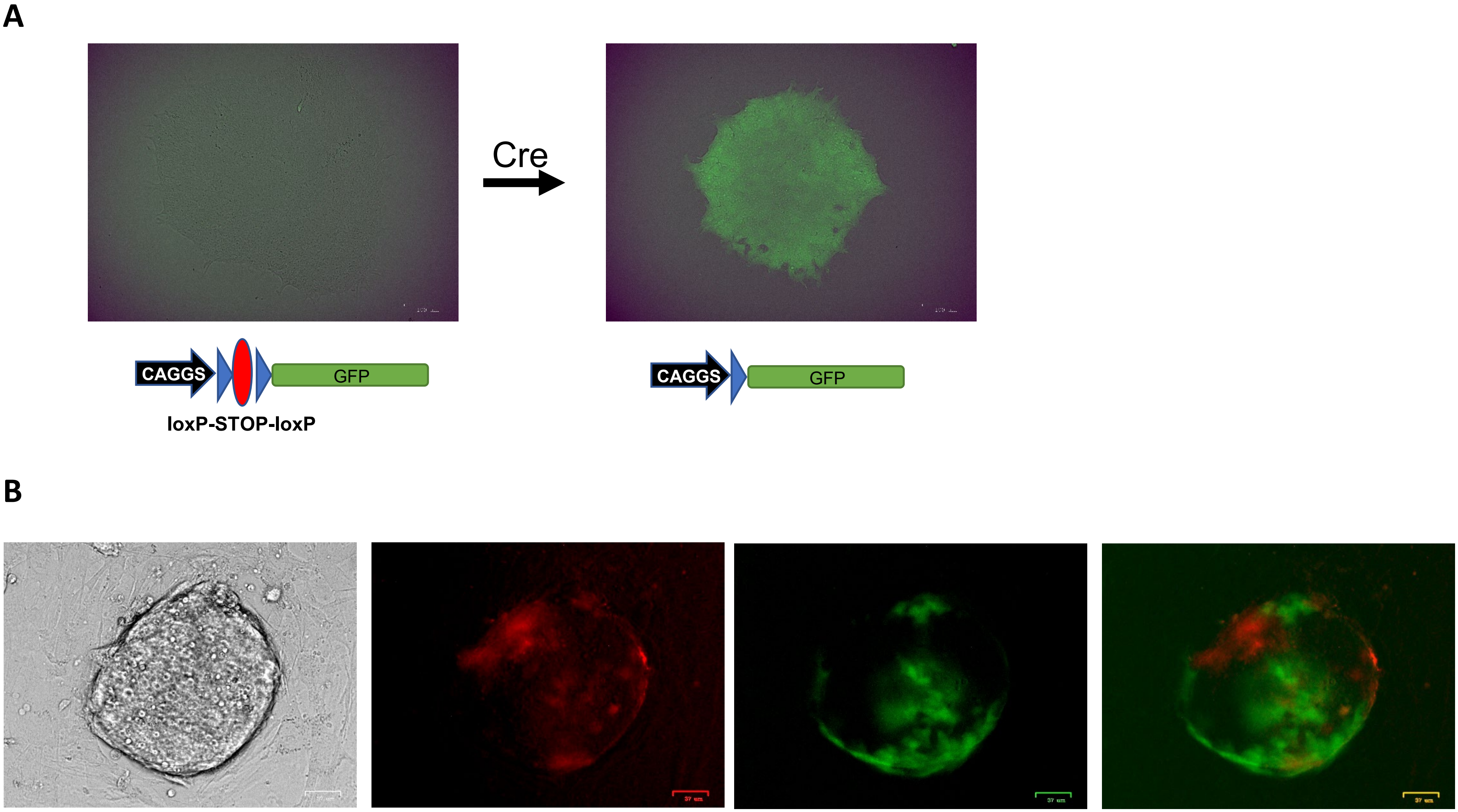

Supplementary Figure 1: A) Human iPS cells with a CAGGS promoter–loxP-STOP-loxP-GFP cassette inserted at the *AAVS1* locus. Following Cre recombinase transfection, the excision of the STOP signal activates GFP expression leading to homogenous expression within the iPS cells. B) Imaging of a mouse ES cell colony harbouring the Traffic Light Reporter inserted at the *Gt(ROSA26)Sor* locus. The cells have been electroporated with an sgRNA targeting the inactive mRFP1 gene with an ssDNA to restore its function. The GFP expression is indicative of NHEJ repair, with a frame-shift of +2 due to an indel restoring the reading frame of the GFP gene, whereas the mRFP1 expression is indicative of HDR repair, with the mRFP1 corrected by successful recombination with the repair ssODN.

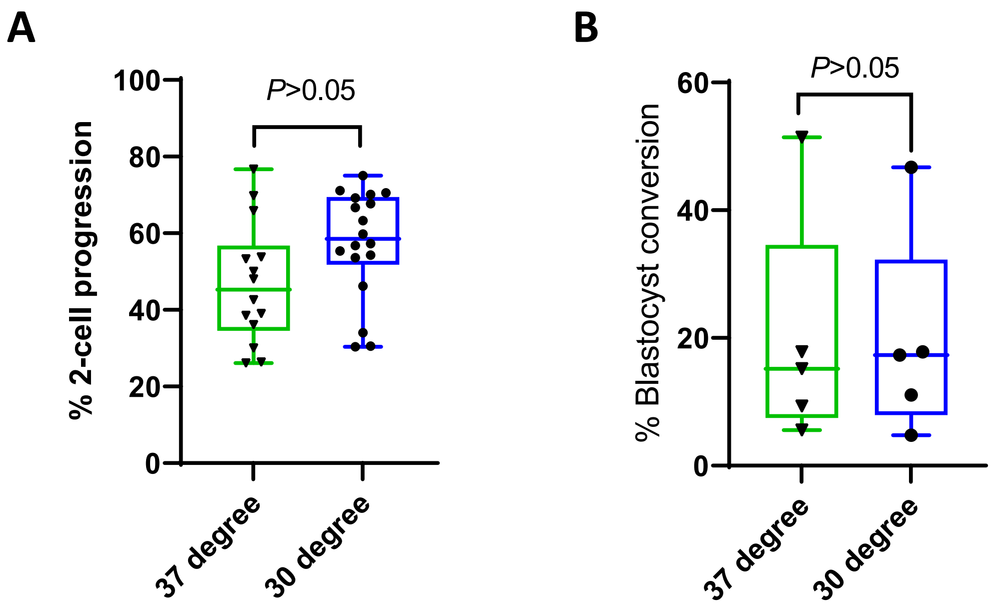

Supplementary Figure 2 – Effects of subjecting 1-cell zygotes to 6-8 hours of 30˚C cold-shock on A) mouse 2-cell development and B) blastocyst development

Target Locus	Rep. colour	Protospacer 5'-3'	Genomic coordinates	Forward Genotyping Primer	Reverse Genotyping Primer
Jcad		GCATGCCTTCAGGCTGACAT	Chr7: 87,073,979-87,142,720 (-)	CTGGCAAAGTCCTGCAGTTG	ACATTGGTCCCTATGGTGGT
Kcnab1		CTCTCACATGGCATCATTGG	Chr3: 64,856,617-65,285,644 (+)	CCCAGTGTCTGAGGGTAGGA	CACCCACGTTCATTCCAGGA
Tyr		GGGTGGATGACCTTGAGTCC	Chr18: 4,634,878-4,682,869 (+)	AGGAGAAAATGTTCTTGGCTGTTTTGT	CTTGTTCCCACAATAACAAGAAAAGTCTGT
TLR		GGCCACGAGTTCGAGATCTA	NA	NA	NA
GFP		CTCGTGACCACCCTGACCTA	NA	NA	NA

Supplementary Table 1: Targets for CRISPR/Cas9 mutagenesis together with their genomic coordinates and the primer sequences used for genotyping.

Target Locus	Rep. colour	Template length (nt)	Template sequence	Restriction site
Jcad		139	CAAAGGTTTAGTCCGTTATCATTATGGTAGGAAGCATGCCTTCAGGCTGAAGCTTATAACTTCGTATAATGT ATGCTATACGAAGTTATCATTGGAGCAGTAGCTGAAAACTACATCCTGATCCTTAGGTGGACAGACA	HindIII
Kcnab1		200	AGGTTGCTGAACGGCTGATGACAATTGCCTACGAAAGTGGAGTTAATCTCTTCGACACAGCTGAGGTCTtT GCTGCTGGGAAGTAAGTCAGAACAAGTTTTTAGCTCTCACATGGCATCATTGGTaagcttGGAAGCAAGAGG GTGTGCTCAAACATTGCTGTGGCATTGGCAAGGAGGGACTGCTCTTCTTGTACATAT	HindIII
Tyr		139	CCAGGATATCCTTCTGTCCAGTGCACCATCTGGACCTCAGTTCCCCTTCAAAGGGGTCGACGATCGTGAAA GCTGGCCCTCTGTGTTTTATAATAGGACCTGCCAGTGCTCAGGCAACTTCATGGGTTTCAACTG	PvuI
TLR mRFP1		150	AAGGTGCGCATGGAGGGCTCCGTGAACGGCCACGAGTTCGAAATTGAGGGCGAGGGCGAGGGCCGCCCC TACGAGGGCACCCAGACCGCCAAGCTGAAGGTGACCAAGGGCGGCCCCCTGCCCTTCGCCTGGGACATCC TGTCCCCTCAG	NA
GFP-to-BFP		138	CCTGAAGTTCATCTGCACCACCGGCAAGCTGCCCGTGCCCTGGCCCACCCTCGTGACCACCCTGAGCCACG GCGTGCAGTGCTTCAGCCGCTACCCCGACCACATGAAGCAGCACGACTTCTTCAAGTCCGCCATGCC	NA

Supplementary Table 2: ssODN repair templates and the diagnostic restriction enzyme site used for detection

Gene ID	Condition	Inclusion of HDR template	No. harvested	No. used	No. survived	No. 2-cell	% 2-cell	No. blastocysts[h]	% Blast/ survived	% Blast/ 2-cell	HDR	Mut	Total analysed
Jcad	37°C	Yes	70	70	63	44	70%	36	57	82	6	31	35
Jcad	30°C	Yes	60	60	56	42	75%	28	50	67	10	26	28
Kcnab1	37°C	Yes	60	60	54	23	43%	9	17	39	4	9	9
Kcnab1	30°C	Yes	60	60	54	36	67%	9	17	25	8	9	9
Kcnab1	37°C	Yes	90	90	90	48	53%	16	18	33	3	12	16
Kcnab1	30°C	Yes	90	90	90	64	71%	16	18	25	3	13	16
Tyr	37°C	Yes	105	105	105	41	39%	16	15	39	0	16	16
Tyr	30°C	Yes	75	75	75	43	57%	13	17	30	3	13	13
Tyr	37°C	Yes	130	130	125	33	26%	12	10	36	5	12	12
Tyr	30°C	Yes	130	130	125	38	30%	12	10	32	8	12	12

Supplementary Table 3: Production and genotyping summary data for the generation of blastocysts using CRISPR/Cas9 reagents and ssODN delivered by electroporation under normal (37°C) or cold-shock (30°C) conditions.

Gene ID	Condition	Inclusion of HDR template	No. harvested	No. used	No. survived	No. 2-cell	% 2-cell	No. of transfers*	Pups born %<br>No. pups/ no.transferred(%)	White	Chimera	Black
Tyr	37°C	Yes	239	239	235	30	13%	3	7/70 (10%)	6	1	0
Tyr	30°C	Yes	321	321	291	99	34%	4	16/138(11.59%)	14	0	2

Supplementary Table 4: Production and genotyping summary data for the generation of live pups using CRISPR/Cas9 reagents and ssODN delivered by electroporation under normal (37°C) or cold-shock (30°C) conditions.
